# A Minimalistic Technique for Neural Tissue Preservation and Neuroanatomical Education: Quantitative Study of the Elnady Technique on Human Cadaveric Specimens

**DOI:** 10.7759/cureus.31588

**Published:** 2022-11-16

**Authors:** Sheantel Reihl, Yelin Kim, Derek Harmon, Ivan H El-Sayed, Adib Abla, Roberto Rodriguez Rubio

**Affiliations:** 1 Department of Neurological Surgery, University of California, San Francisco, USA; 2 Department of Neurological Surgery, University of California Berkeley, Berkeley, USA; 3 Department of Anatomy, University of California, San Francisco, USA; 4 Department of Otolaryngology-Head and Neck Surgery, University of California, San Francisco, USA

**Keywords:** medical education, preservation, cadaveric dissections, neuroanatomy, technique, alternative, plastination

## Abstract

Cadaveric shortages have been a challenge to anatomy education; as access remains low in many parts of the world, institutions are relying on plastinated specimens. Plastination typically requires the use of complex equipment and patented chemicals. While models solve cost and toxicity issues, in neuroanatomical education, the rigidity prevents deep-brain structure exploration and visual-spatial learning. The Elnady technique (ET) developed by Dr. Fawzy Elnady is an alternative method that solves the limitations of traditional plastination while maintaining the advantages previously developed in animal models.

The superficial temporal artery (STA), brain stem, cerebellum, right hemisphere, and latex-injected cortex were previously embalmed, drained of their original solution, dehydrated in acetone baths, and hydrometer readings were taken. Specimens were placed in a glycerol bath, immersed in cornstarch and cured. Quantitative and qualitative data of weight (grams), size (millimeters), color, texture, and odor were obtained before and after processing the samples.

Overall, specimens showed a change of 6.5% in weight, 8.5% in height, 4.8% in width, and 8.9% in length (millimeters) after the preservation process. The products had pliable texture, no change in color grossly and no detectable odor. The measurement of weight (grams) ranges from 0-15.7%, height from 0-12.3%, width from 0-11.1%, and length from 1.7-5.9%.

The ET is an effective method for the preservation of human cadaveric specimens that produces quality samples from embalmed specimens. Preservation can be done without patented chemicals and special storage methods are usually required for plastination. It is an ideal technique for basic and/or low-resource settings and could resolve expenses related to acquiring and maintaining cadaveric brain specimens.

## Introduction

In recent years, there has been a major push in medical education to explore new methods of teaching neuroanatomy beyond the wet dissections of cadaveric specimens [1-3]. This is due to the increasingly growing problem of cadaveric shortages in several parts of the world like Canada, Sweden, Greece, South America, and North America. This, in turn, impinges on the quality of anatomical education that can be provided [4-6]. Currently, there is a strain on the accessibility to cadavers in respect to the dwindling numbers over the recent years, and many institutions have been utilizing plastinated specimens [7-9]. However, while plastinated models provide a generic overview structure, the rigidity of these models hinders the deep exploration of neuroanatomical structures and the visual-spatial learning that is imperative to fully grasp the underlying anatomy [10,11]. Cadaveric dissection is expensive, time-consuming, and in many medical schools that have to accommodate 200-400 students each year, it is quickly becoming a luxury they cannot afford to have [12]. Further, in order to minimize costs, they must hold onto these specimens for many years, even as the educational quality of the specimens rapidly declines. Lately, there has been a push to move away from wet dissections as a whole to circumvent its challenges. Many institutions have been experimenting with prosections-only anatomy labs, three-dimensional (3D) educational modules and atlases, and other online tools [2,7,13]. However, early research has been inclusive of the utility of these alternative methods of teaching.

Plastination, in itself, is not a new technique. It is a broad term for preservation that varies in materials and techniques used across the world [10,14,15]. Still, we typically expect it to produce hard specimens that are mostly associated with glass cases in museums [16]. Yet, plastination as a tool has not been widely utilized in medical education and presents a possible solution to the predicament faced in teaching anatomy. Some of the most common forms of plastination include the use of highly toxic compounds with strong odors and putting the learner at risk with the known carcinogenic effects of formaldehyde exposure [17]. Most forms of plastination create hardened specimens that cannot be manipulated nor properly examined, preventing the learner from some of the small yet significant details in neural structures. Furthermore, the procedure to produce these plastinated specimens requires the use of patented chemicals and equipment that are not only expensive to acquire but require specialized training [18-20].

The Elnady technique (ET) is a new method of plastination developed by Fawzy Elnady at Cairo University in the preservation of animal specimens as part of the school of veterinary medicine [21,22]. It requires non-patented, common laboratory reagents and household goods, with the entire procedure able to be carried out at room temperature on a lab bench [21]. An evaluation of this method in human specimens, particularly neural tissue, has not yet been investigated. Nevertheless, the use of the ET presents a unique method to maintain structural integrity, achieve the desired hands-on experience, lower the health risks to students, as well as minimize the costs to institutions.

This study aims to investigate the application of dry, room-temperature preservation of human neuroanatomical specimens under a minimalistic environment and to analyze the macroscopic structural modifications on the samples after utilizing the procedure outlined in the Elnady technique. This work was presented at the Experimental Biology Meeting held on 23 April 2018.

## Materials and methods

Prior to processing the samples, specific measurements of five specimens: a human superficial temporal artery (STA), brainstem, right cerebellar hemisphere, right brain hemisphere, and latex-injected brain with an axial cut at the level of the basal ganglia were each taken, including the weights (grams), heights (millimeters), widths (millimeters), and lengths (millimeters) along anatomically important axes pre-and post-treatment. The entirety of the procedure was performed on a lab benchtop, without a hood, in a well-ventilated, temperature-stable lab room. The materials used in this experiment were easily obtainable as they were commercially available to buy. This experiment did not entail directly working with patients themselves. Instead, the specimens used in these experiments had all been previously fixated with formaldehyde-based mixtures at the University of California San Fransico (UCSF) Willed Body Program before their extraction from embalmed cadaver donors (See Table 1 for the precise composition of the preservatives used). Additionally, a right brain hemisphere underwent white matter dissection after preparation with the Klingler method [23]. No IRB/ethics committee approval was required for this study. 

**Table 1:** Cost and sources of the materials used in the Elnady technique for the plastination of human neural tissue.

	Item	Cost	Notes
Step 1	1. Formalin	$163 (Fisher Scientific)	10% Fisher Scientific
	2. Large containers for baths	$10 (Amazon)	Rubbermaid, 500mL (3ct), 3L (1ct)
	3. Syringes	$15.15 (Grainger Industrial Supply)	Already in lab
	4. Cannula	$27 (Ambler Surgical)	Already in lab
	5. Distilled water	-	4L Reagent grade
Step 2	6. Colored latex/epoxy dye	$5.75 (Stone coat countertops)	Already in lab
Step 3	7. Pure (100%) acetone	$47.24 (Fisher Scientific)	1L HPLC>99.9%
	8. Hydrometer	$53.63 (Fisher Scientific)	Already in lab
Step 4	9. Sieve	$17.36 (eBay)	12" Stainless steel
	10. Glycerin	$38 (Grainger)	4L ACS grade; 99 to 100%
Step 5	11. Tissue paper	$1.34 (Kleenex)	Already in lab
	12. Ziplock bags	$12.45 (Amazon)	X-Lg 96ct
	13. Cornstarch powder	$73.13 (Amazon)	50lb bag
	14. Soft brush	$5.73 (eBay)	Dusting brush

Each specimen was removed from its 10% formaldehyde-based container, washed with water, and allowed to drain on absorbent cloths. It should be noted that to minimize the risk of tissue damage, specimens were not completely dry before being dehydrated, and thus pre-treatment weights include a considerable amount of retention of the formaldehyde solution. Each specimen was then added to a pure (100%) acetone bath in a closed container for dehydration at room temperature. The amount of acetone added ranged (200ml-3.8L) by specimen size to accomplish a level of about 5cm of height above the highest point of the specimen lying flat in the container. The concentration level of acetone was measured with a hydrometer (Fisher Scientific Company LLC, Hanover Park, IL, USA) twice daily until measurements showed consistent acetone concentration readings of 99% for a 48-hour period. The time to reach this level ranged for each specimen, according to size, from 3-9 days.

After consistent readings, specimens were removed from the acetone bath and again allowed to drain at room temperature on absorbent cloths through a sieve, typically for less than five hours. Impregnation was completed with a pure glycerin liquid under room pressure through submersion. Each specimen was placed in a separate glycerin bath with weights to ensure complete submersion. Specimens remained submerged for a period of two weeks.

After removal from the glycerin bath, each specimen was pressed down thoroughly using tissue paper and allowed to drain off excess glycerin. Each specimen was then transferred into plastic bags 2-3x the size of the specimen filled with cornstarch powder. Excess glycerin and cornstarch clumped together were periodically removed, and fresh cornstarch was added. The curation period lasted 3-10 days, depending on specimen size with multiple powder changes. After completion, each specimen was rinsed with tap water to remove excess powder and allowed to air dry on a countertop.

## Results

**Figure 1:**
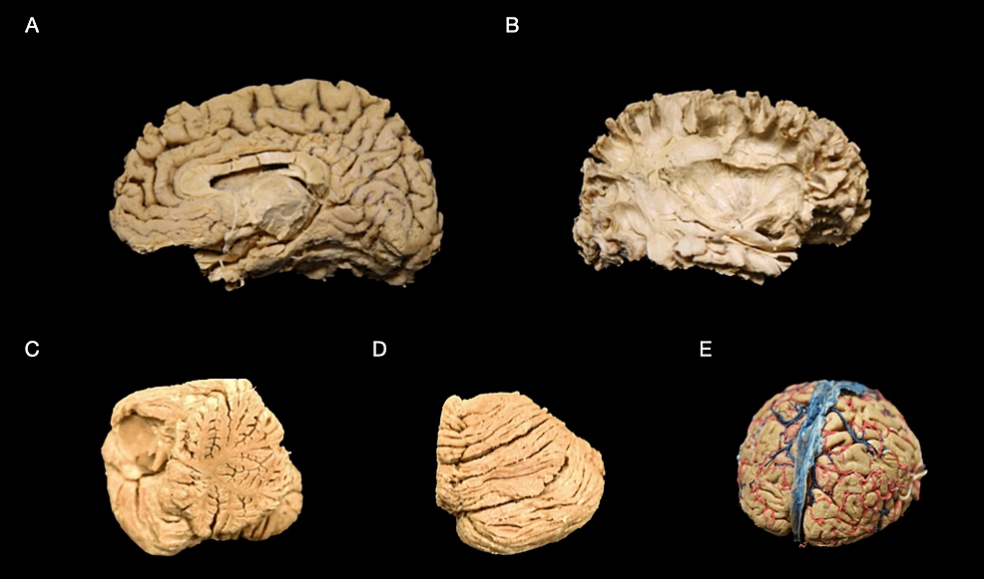
Specimens Pre-treatment using the Elnady technique. (A) Medial surface of the right hemisphere depicting perisylvian network after white matter dissection following the Klingler method pre-treatment. (B) The lateral surface of the right hemisphere depicting the perisylvian network after white matter dissection following the Klingler method pre-treatment. (C) Medial view of the right cerebellum hemisphere pre-treatment. (D) The superior view of the right cerebellum hemisphere pre-treatment. (E) Superior surface of the plastinated hemisected (axial) cortex, previously fixated in formaldehyde and injected with red and blue latex dyes, prior to treatment using the Elnady technique.

**Figure 2:**
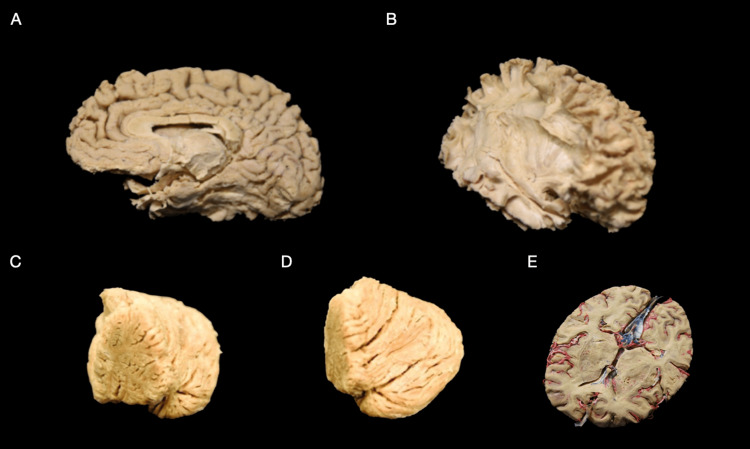
Specimens three weeks post-treatment. (A) Medial surface of the right hemisphere depicting the perisylvian network after white matter dissection following the Klingler method post-treatment. (B) The lateral surface of the right hemisphere depicting perisylvian network after white matter dissection following the Klingler method post-treatment. (C) Medial view of the right cerebellum hemisphere post-treatment. (D) The superior view of the right cerebellum hemisphere post-treatment. (E) The inferior surface of the plastinated hemisected (axial) cortex, previously fixated in formaldehyde and injected with red and blue latex dyes, post-treatment using the Elnady technique.

**Figure 3:**
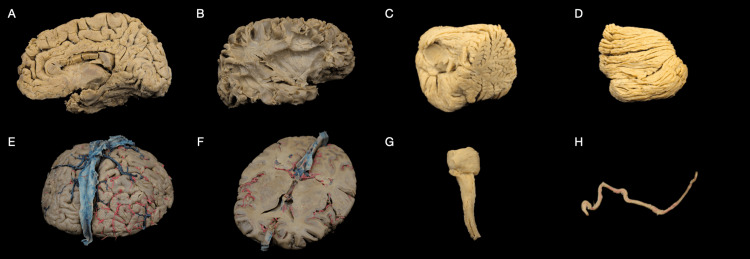
Specimens four years post-treatment. (A) Medial surface of the right hemisphere depicting the perisylvian network after white matter dissection following the Klingler method post-treatment. (B) The lateral surface of the right hemisphere depicting the perisylvian network after white matter dissection following the Klingler method post-treatment. (C) Medial view of the right cerebellum hemisphere post-treatment. (D) The superior view of the right cerebellum hemisphere post-treatment. (E) The superior surface of the plastinated hemisected (axial) cortex, previously fixated in formaldehyde and injected with red and blue latex dyes, post-treatment using the Elnady technique. (F) The inferior surface of the plastinated hemisected (axial) cortex, previously fixated in formaldehyde and injected with red and blue latex dyes, post-treatment using the Elnady Technique. (G) Brain stem post-treatment. (H) Superficial temporal artery (STA) post-treatment.

The specimen measurements were taken three weeks after the first day of treatment (September-October 2017). The final products had virtually no change in color that could be noted grossly and had a soft, pliable texture and no residual odor from their prior formaldehyde preservation. On average, the specimens altogether showed a change of 6.5% in weight, 8.5% in height, 4.8% in width, and 8.9% in length. Pre-and post-treatment measurements, as well as percentage changes for the STA, brainstem, right cerebellar hemisphere, right brain hemisphere, and latex-injected brain with an axial cut, are reported in Table* *2. A long-term follow-up morphometric measurement was taken four years after treatment with the ET (September 2021). Photographic evidence was taken at different stages throughout the preservation process (Figures 1-3).

**Table 2:** Morphometric data from specimens pre-and post-plastination treatment with the Elnady technique.

	Weight Pre>Post Treatment (g)	Weight Pre>Post Treatment 2021 (g)	Percent Δ (%)	Percent Δ 2021 (%)	Height Pre>Post Treatment (mm)	Height Pre>Post Treatment 2021 (mm)	Percent Δ (%)	Percent Δ 2021 (%)	Width Pre>Post Treatment (mm)	Width Pre>Post Treatment 2021 (mm)	Percent Δ (%)	Percent Δ 2021 (%)	Length Pre>Post Treatment (mm)	Length Pre>Post Treatment 2021 (mm)	Percent Δ (%)	Percent Δ 2021 (%)
Superficial Temporal Artery (STA)	0.2>0.2	0.2>0.2	0	0	0.6>0.6	0.6>0.3	0	50	2.4>2.4	2.4>2.3	0	4.2	75.8>74.5	75.8>73.8	1.7	2.6
Brain Stem	8.5>7.8	8.5>6.7	8.2	21.2	22.0>19.3	22.0>17.6	12.3	20	26.2>23.3	26.2>23.2	11.1	11.4	68.2>64.2	68.2>63.1	5.9	7.5
Cerebellum	39.6>33.4	39.6>26.2	15.7	33.8	39.0>38.2	39.0>37.8	2.1	3.1	44.0>43.2	44.0>42.2	1.8	4.1	48.5>46.3	48.5>44.5	4.5	8.2
Right Hemisphere	223.5>218.3	223.5>143.3	2.3	35.9	50.9>47.8	50.9>36.9	6.1	27.5	80.9>78.3	80.9>77.4	3.2	4.3	140.7>135.5	140.7> 130.8	3.7	7.0
Latex Injected Cortex	652.0>	652.0>443.0		32.1	79.8>	79.8>61.6		22.8	121.9>	121.9>116.0		4.8	153.2>	153.2>137.2		10.4

## Discussion

The findings in the human neural tissue preservation for neuroanatomical specimens exhibited minimal shrinkage, maintained the quality of color and texture, and showed that it was safer to handle with the reduction in formaldehyde exposure for the user. Utilization of this method on five of our human cadaveric specimens--STA, brainstem, right cerebellar hemisphere, right brain hemisphere, and brain with axial cut, all of which had been previously fixated in 10% formaldehyde-based solutions--resulted in malleable specimens that demonstrated a minimal loss in weight, size and coloration and had neutral odor upon completion. The soft preservation of these specimens achieved through the use of the ET highlights a new direction for the preservation of human specimens in medical education [21]. The procedure was successfully performed at room temperature and pressure throughout, with the use of minimal laboratory equipment, non-patented chemicals, and store-bought impregnating and curative agents. Furthermore, executing the procedure required minimal laboratory space, and minimal technical skills and it could be completed for large brain sections in less than three weeks.

Plastination is often discounted as an appropriate tool for preparing specimens for medical education. It is typically associated with hard products that cannot be manipulated to explore deep structures. Further, plastination usually requires large laboratory space to carry out the vacuum, high-pressure protocols, exorbitantly priced patented polymers and many months until completion. For these reasons, it is often not a viable option for low-resource laboratories and medical schools, even when cadaveric specimen supplies are low [24]. Some limitations of this study are that all specimens treated had been previously fixated and stored for more than a year. Thus, we cannot find evidence to support this method in fresh specimens. In addition, due to their previous-stored state, complete water drainage before preservation was not possible for measuring pre-treatment weights. However, our findings from this study demonstrate that preservation can be an effective tool for producing soft specimens that do not sacrifice any desirable qualities achieved in the typical formaldehyde preservation method. The original ET study found that produced miscellaneous specimens of animal parts were as realistic in terms of their durability, odor, texture, and flexibility as their original state [20]. Likewise, in this experiment, the transposition of the technique to human brain structures showed results indicating that this is a viable technique that may be conducive to enhancing medical anatomical education.

Within our research, a longitudinal observation was carried out to show the long-term preservation results of the four specimens. Our measurements showed that between 2017 and 2021, the specimens had maintained a physical appearance very similar to when it was first preserved. In the case of the brain stem, there was a 0.3% change in width between the post-preserved specimens between 2017 and 2021. This is a significant result because in those four years the specimen preserved its physical appearance almost fully which would be conducive in medical learning settings for long-term usage. However, while most specimens in other measurements in the aspects of weight, height, width, and length did not present vast differences, it is noted that in the superficial temporal artery (STA), there was a 50% decrease in height. This can be attributed to the structure of the STA naturally because it is tortuous and in its physical state, it is already very thin.

Several modifications and adaptations to the modern conventional plastination method initially described by von Hagens have been developed in recent years [15]. It was noted that most research applications utilized the sheet plastination technique; however, a realistic estimate for the establishment of a sheet plastination laboratory approximates to US $50,000 for equipment [15]. Another paper by Ottone et al. contributed to the development of a plastination technique using silicone at room temperature [19]. The analysis of the results showed that the volume and shape of the cerebral hemisphere stayed the same as at the beginning of the process, the texture and color were preserved very similarly to their original state, and the specimens were dry and rigid. Our study was done to analyze the long-term modifications of specimens. There have been other studies that investigated knee joint specimens to see the difference between new and old plastinated specimens [25]. Our study however, successfully expounded on whether those newly plastinated specimens would deteriorate over time to evaluate the feasibility of long-term usage [25].

There have been notable research done within this field to address the concerning issues with the diminishing number of cadavers for anatomy study. In Masuko et al., there was a similar study done on the female human body as a whole. Likewise, in the current paper, this study produced results of soft and flexible cadavers, collecting measurements 150 days after the embalming process was done [26]. Our current study concentrates deeply on the brain structure in itself with its intricate complexities. To date, there have not been any papers that take specific measurements of the specimens in terms of pre- and post-treatment and even a four-year follow-up. It is important to emphasize that being able to preserve this organ in a way that visual-spatial learning can be done is imperative to the nature of the study and future research that can be carried out with these ET-preserved models.

Future directions for continuing to validate this method would include comparative studies using previously described plastination techniques, histological analyses of the samples, and the application of the technique at a larger scale on specimens with multiple tissue characteristics. Additionally, new technologies such as 3D scanning and 3D data analysis will potentially help us to better understand the volumetric and structural changes of the specimens after long-term preservation. Due to the nature of this method being fairly newer, further investigation would also include the examination of the durability of the preserved specimens in comparison to the conventional specimens preserved through plastination, even though we can observe through the original publication of the minimalistic technique described by Elnady that animal parts were successfully preserved for over a year. 

Study limitations

Our study did not run a comparative and double-blind study. Future directions would be to have subjective evaluation of the specimens in terms of its durability, flexibility, and color change from other students and faculty members. Overall, the specimens have shown great potential as an alternate path to expanding access to cadaveric models in medical schools and anatomical laboratories at a low cost while eliminating all of the major disadvantages associated with plastinated specimens. Additionally, the lack of photogrammetric evidence of the different stages of specimens might be an obstacle to having a clear visual representation of the dimensional modifications even though objective morphometric data were provided.

## Conclusions

In this study, we investigated the use of a low-cost Elnady preservation technique. Our primary goal was to qualitatively and quantitatively evaluate the utility and effectiveness of this technique on human cadaveric neural tissues in the laboratory setting. In line with the original intention, this study was a longitudinal morphometric evaluation to investigate the long-term durability of the specimens for usage. Further, we sought to demonstrate the practicality of executing this method in preparing neuroanatomical specimens. Our results support the use of the ET for producing high-quality neuroanatomical specimens with minimal shrinkage, texture, and color changes that are pliable and easy to work with and lack odorous smells. In addition, itemization of the equipment and materials needed for this preservation technique demonstrates that this method can be easily prepared in low-resource laboratory space, under room temperature and pressure conditions, with low-toxicity materials and minimal costs.
